# Functionalization of Gold Nanoparticles by Inorganic Entities

**DOI:** 10.3390/nano10030548

**Published:** 2020-03-18

**Authors:** Frédéric Dumur, Eddy Dumas, Cédric R. Mayer

**Affiliations:** 1 Aix Marseille Univ, CNRS, ICR, UMR 7273, F-13397 Marseille, France; 2 Institut Lavoisier de Versailles, UMR CNRS 8180, Université de Versailles Saint-Quentin-en-Yvelines, F-78035 Versailles, France; eddy.dumas@uvsq.fr; 3 Laboratoire LuMin, FRE CNRS 2036, CNRS, Université Paris-Sud, ENS Paris-Saclay, Université Paris-Saclay, F-91405 Orsay CEDEX, France; 4 Département de Chimie, UFR des Sciences, Université de Versailles Saint-Quentin-en-Yvelines, F-78035 Versailles, France

**Keywords:** Au°-Nanoparticles, *d* and *f* metallic coordination and organometallic complexes, SiO_2_ coating, *p*- and *s*-block elements

## Abstract

The great affinity of gold surface for numerous electron-donating groups has largely contributed to the rapid development of functionalized gold nanoparticles (Au-NPs). In the last years, a new subclass of nanocomposite has emerged, based on the association of inorganic molecular entities (IME) with Au-NPs. This highly extended and diversified subclass was promoted by the synergy between the intrinsic properties of the shell and the gold core. This review—divided into four main parts—focuses on an introductory section of the basic notions related to the stabilization of gold nanoparticles and defines in a second part the key role played by the functionalizing agent. Then, we present a wide range of inorganic molecular entities used to prepare these nanocomposites (NCs). In particular, we focus on four different types of inorganic systems, their topologies, and their current applications. Finally, the most recent applications are described before an overview of this new emerging field of research.

## 1. Introduction

In nanosciences, gold nanoparticles (Au-NPs) constitute a specific field of research, as chalcogenides or metallic oxides. Gold has always been a source of interest, which dates from antiquity [1]. Since the emergence of modern chemistry, Au-NPs chemistry has known two major steps in its development. In 1951, Turkevich presented the first synthesis of gold nanoparticles in aqueous solution, resulting in polydisperse nanoparticles [2]. In 1994, Brust described a unique and simple method to obtain well-monodispersed and functionalized NPs, by use of a two-phase liquid-liquid system—since, commonly named “*Brust method*” [3]. Starting from this date, functionalization of gold nanoparticles has known an ever-increasing research effort. This development has notably been supported by the intrinsic physicochemical properties of Au-NPs playing a key role in numerous fields of both fundamental and applied research. In particular, Au-NPs display fascinating electronic and optical properties [4,5,6,7,8]. Their abilities to ensure controllable interactions with various organic electron-donating groups have also largely contributed to their developments. Nowadays, use of Au-NPs has been extended to domains ranging from biomedical applications [9,10,11,12] to electronics [13,14], as a result of the great advances in their functionalizations and their stabilizations. Since the pioneering works concerning the functionalization processes of gold surfaces, the nature of the functionalizing agents is now nearly unlimited, but strongly depends on targeted applications.

In this review, we focus our attention on inorganic molecular entities (IME) as functionalizing agents. Notions of stabilization of gold nanoparticles and functionalization of Au-NPs are preliminary presented. Then, four types of IME are detailed with their topologies and potential applications: bloc *p*, metallic oxoclusters, organometallic complexes, and coordination complexes. Finally, we conclude with significant applications concerning these nanocomposites and their futures.

## 2. Notions on Stabilization of Gold and Silver Nanoparticles

### 2.1. Role and Nature of the Stabilizing Agent

**Scheme 1:**
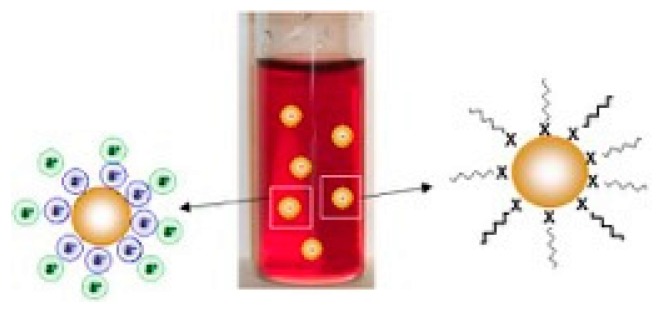
Both processes of nanoparticles (NP) repulsion by electrostatic or steric repulsion, induced by charged molecules or long chains, respectively.

Nowadays, preparation of gold nanoparticles is relatively easy, due to the different reduction processes at our disposal for their syntheses. To introduce a specific function—or to get size-monodispersed NPs in a desired solvent at a desired concentration with an expected morphology—knowledge of the role and the nature of the stabilizing agent is crucial. Stabilization of colloidal suspensions results from two specific processes: the first is based on steric repulsion, induced by long alkyl chains grafted onto the gold surface [15], and the second results from electrostatic repulsions resulting from the use of charged ligands [16], as shown in the Scheme 1.

**Scheme 2:**
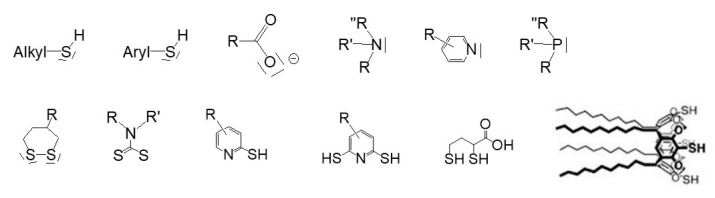
Nature of the anchoring groups.

Concerning the stabilization of colloids by steric repulsions, the interaction of the stabilizing agent with the NPs surface is usually ensured by use of electron donating groups. Different anchoring groups have been employed in the literature [17], but the most common ones are, without contest, alkylthiol or arylthiol groups [17] (Scheme 2). The sulfur atom is the element that ensures the strongest interaction with gold atoms. Other functions have also been used to graft a stabilizing agent onto the NPs surface: phosphine [18,19,20,21], amine [22], pyridine [23], and carboxylate [24] (See Scheme 2). To prevent undesired desorption of a stabilizing agent, an efficient method consists of using molecules possessing at least two electron-donating groups, such as disulphide [25], dithiocarbamate functions [26], or trithiol species [27]. Mixed donating groups such as mercaptopyridine, [28] mercaptothiadiazole [29], or lipoic acid derivatives [30] that possess two anchoring groups also efficiently ensure a strong interaction with the NP surface. These polydentate ligands render functionalized NPs effectively more stable towards ligand exchange.

To control the size of spherical NPs, the best process consists of adjusting the ratio between the Gold (III) salt and the stabilizing agent [31,32]. High concentrations of well-monodispersed NPs are generally obtained with long alkyl chains (length > C_8_H_17_) [33,34] or with polymers [35]. Small NPs with diameters around 1.5 to 3 nm are also obtained in these conditions. Preparation of large or small and monodispersed Au-NPs remains still a challenge at present. However, Wei et al. succeeded to synthesize a resorcinarene tetrathiol that provided very monodispersed NPs with a large size [36]. Another way to get a similar result consists of using hydrothermal conditions from Au-NPs seeds [37].

The monodispersity and size of NPs are currently controlled by the initial molar ratio of the gold (III) salt and the stabilizing agent, due to the fact that both parameters are highly dependent. When a strong coordination with gold atoms is ensured by the ligand—as in the case of sulfur atoms—all gold-clusters are covered by the ligands, immediately stopping the growth of the different crystalline faces. Small particles are thus obtained. On the other hand, when an electron-donating group ensuring a weak interaction with the gold surface is used, desorption of the ligand can occur, allowing the growth of a crystallization face, or the aggregation of particles. Polydispersed particles and aggregates are thus obtained. Other parameters can have a non-negligible influence on both the surface and morphology, such as the topology of the ligand, the solvent, the temperature, the strength of reducing agent, or the pH, in the case of an aqueous medium. Due to the influence of these different parameters, the use of a new stabilizing agent implies a preliminary control of these different points (ratio, nature of the solvent, temperature of the medium, nature, and ratio of the reducing agent).

### 2.2. Synthetic Methods

Gold nanoparticles are used in a multitude of applications ranging from biomedicine, to catalysis, optics, electronics, and 1D-3D-assemblies. With few exceptions, the most important prerequisite for all these applications remains the preparation of highly uniform-sized monodispersed NPs and well-stabilized NPs. A wide range of applications such as optical and electrochemical applications, requires monodispersed NPs of controlled size to analyze single NPs. Moreover, the control of self-assemblies of NPs also necessitates to have very monodispersed NPs [38].

Various processes may be used to prepare Au-NPs. The dominant synthetic approach consists of the reduction of a gold (III) salt (currently AuCl_4_^−^) in the presence of a stabilizing agent by use of a reducing agent (classically, NaBH_4_). Sometimes, the stabilizing agent and the reducing agent may be the same reactant. This is the case when citrate anions [2] or aniline is used: the reducing agent stabilizes the particles [22]. Two variants of these two synthetic procedures are commonly used to prepare particles. Thus, the reduction process can occur in a one-phase system (all reactants are solubilized in the same solvent) [39] or in a two-phase system (where the reduction is done at the interface between both solvents, following a preliminary transfer of the AuCl_4_^−^ anion in the same solvent as the stabilizing agent) [40]. Other modifications of these initial procedures allow, for instance, the control of the morphology of NPs: such is the case when CTAB (cetyltrimethylammonium bromide) is used as a stabilizing agent, resulting in the formation of nanorods [41]. Different sources of reduction may also be used instead of the usual chemical reducing agent, NaBH_4_. (c)-irradiation is thus able to produce NPs of different morphologies, determined by the experimental process [42]. Electrochemical [43] and photochemical (by UV irradiation) [44] procedures are other alternatives for the reduction process [45]. More recently, ultrasonication has also been used as a reduction method for gold ions [46,47].

## 3. Prerequisite for Functionalizing Agents

### 3.1. Definition

**Scheme 3:**
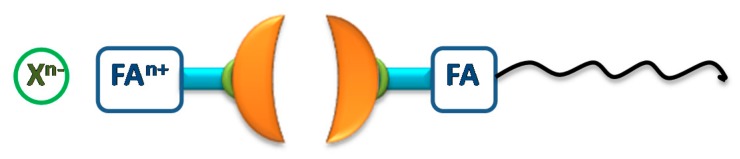
Functionalization of the gold core.

The term “functionalization” is not precisely defined in the literature. Different notions can be underly this term: definition of this term, role (interest of the functionalization), or grafting mode (way how to functionalize the particles). Hence, in the case of a metallic oxocluster, functionalization simply means to link in a covalent way an organic function to the oxocluster [48]. But in the case of Au-NPs, their existence and stability are mostly due to the organic function, ensuring only a role of stabilizing agent (See Scheme 3). Herein, we define the words, “functionalizing agent” as a molecule or an entity able to exhibit specific properties in addition to the simple ability to stabilize particles. These properties can be a specific organic function (as an alcohol or an amine function or other groups) [49,50], an additional physico-chemical properties (such as optical or electrochemical properties) [51], or lead to a specific application such as in biomedicine by grafting of a chemotherapeutic agent [52].

The possibility to combine intrinsic properties of the coating agent (functionalizing agent) with the intrinsic properties of the gold core can generate innovative advanced materials with promising applications expected in many fields, including optics, electronics, catalysis, sensors, biology, and others. Another important point concerns the properties of the functionalizing agent. In order to keep the stability of NPs after functionalization, the functionalizing agent must induce sufficient repulsions between NPs as a simple stabilizing agent could do. Another important point also consists of avoiding a modification of the size and the morphology of the final NPs after functionalization of preformed nanoparticles by ligand exchange.

In the case of the direct synthesis of gold nanoparticles in the presence of the functionalizing agent, the goal is to directly get the most monodispersed NPs and the most stable colloidal solution by optimizing the experimental conditions. In this review, an overview of all inorganic molecular entities (IME) reported as functionalizing agents is presented.

### 3.2. Method to Functionalize Au°-Nanoparticles

**Scheme 4:**
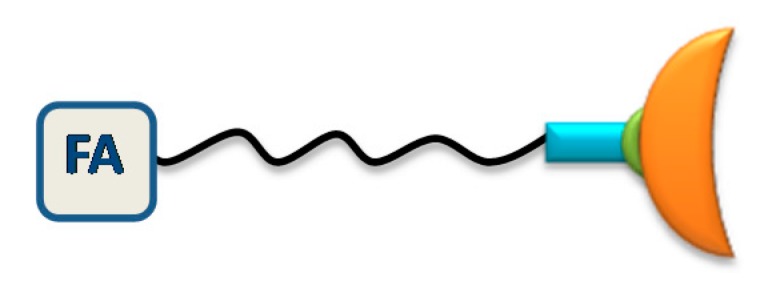
The functionalizing agent (FA) is grafted to the extremity of the long alkyl chain that ensuring steric repulsion.

Introduction of the functionalizing agent onto the gold surface can be realized according to two different procedures. The first process corresponds to direct functionalization by the only use of the functionalizing agent; the second one by use of a mixture of the functionalizing agent with the stabilizing agent. In the last case—and to avoid the formation of two types of NPs (the first batch of NPs stabilized only with the stabilizing agent, *SA*, and the second one with the functionalizing agent, *FA*)—it is necessary to use similar anchorage groups for both agents. In this process, reduction occurs in the presence of *FA* or in the presence of mixed *FA*/*SA* with the desired ratio. Another type of process consists of the post-functionalization of preformed NPs (See Scheme 4).

**Scheme 5:**
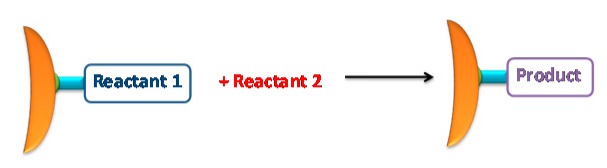
Chemistry directly realized onto prefunctionalized NPs.

This process can occur following two methods: by ligand exchange of *SA* by *FA* [18,53], and by reactivity onto Au-NPs [54]. In the last case, prefunctionalized NPs are synthesized and then the reaction is performed onto the Au°-NP platform. As last point requiring different approaches, the length of the spacer between NPs and the *FA* is of crucial importance (See Scheme 5).

The close proximity of both components can significantly alter the physical properties of *FA* (as the possible quenching of the luminescence of *FA*), or can influence the stability of the final composite. For all these reasons, many studies of the literature used long thiolated alkyl chains, terminated by *AF*. The thiol group ensures a strong interaction with the gold surface and the long alkyl chain induces a good repulsion between NPs [55].

### 3.3. Technical Characterization of Functionalized Au-NPs

**Scheme 6:**
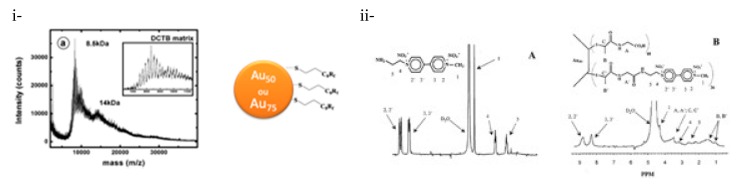
Example of technical methods to characterize smaller gold nanoparticles. (**i**)- by MALDI-TOF mass spectrometry, published in [61], ACS 2007 or (**ii**)- by ^1^H-NMR spectrometry, with figure showing in (**A**), the free ligand and in (**B**), the same ligand surrounding the Au-NPs. From [60], RSC 2003.

Characterization of NPs can be performed using different complementary techniques, each technique bringing its specific information (size, shape, composition of the sample, surface functionalization, charge, etc.). Methods to characterize NPs can also vary, depending on whether the ligand is charged or not. A complete characterization of the particles can only result from the combination of several techniques. One of the most classical techniques to characterize Au-NPs is the transmission electronic microscopy (TEM) for imagery with analysis by X-ray diffraction and depending the sample coupled with energy dispersive X-ray to get a qualitative elementary analysis. Another conventional technique is the UV-visible spectroscopy and for anisotropic forms near infra-red spectroscopy [56]. Gold nanoparticles exhibiting a typical band named resonance plasmon band, which is strongly dependent of the shape, the size, and the nature of the coating agent in the UV-visible, and sometime the NIR region, position of this band can be detected by UV-visible spectroscopy [57]. But in many cases, the resonance plasmon band can be hardly detected due to the fact that *FA* exhibit visible or UV bands superimposing the plasmon band. Another method for an easy control of the size distribution is the dynamic light scattering [58]. When *FA* is charged, zeta potential can constitute a good technique to evaluate the hydrodynamic radius of NPs in solution [59]. ^1^H-NMR spectroscopy has also been used to characterize *FA*-capped Au-NPs (See Scheme 6) [60]. The enhancement of broader bands constituted a proof of coating. As an ultimate technique mainly used for small NPs (<2 nm), mass spectrometry, with the use of specific MALDI-TOF, is a powerful technique to analyze functionalized NPs [61,62].

Finally, other efficient characterization techniques such as Fourier-transform infrared spectroscopy (FT-IR) and surface-enhanced Raman spectroscopy (SERS) can be cited to characterize gold nanoparticles [62,63].

## 4. Functionalization by Inorganic Entities from *P*- Block

Due to the covalent character of the bonds formed by the *p*-block elements of the periodic table, organic molecules are nearly exclusively prepared with these elements, but also with elements coming from columns V to VII. Few inorganic entities based on *p*-block elements have been used to functionalize Au-NPs and most of them are obtained under the form of clusters, like fullerenes, carborane clusters or others. In this part, we describe in a first paragraph the functionalization of Au-NPs by these clusters and in a second one, we focus our attention on silica coatings to design functionalized Au°*_core_*/SiO_2*shell*_ nanocomposites. We end this subpart with a new area in full expansion that concerns the association of carbon nanotubes to Au-NPs.

### 4.1. Molecular Clusters

In the literature, three main molecular clusters have been extensively studied: fullerenes (C_60_), carborane clusters, and polyhedral oligomeric silsesquioxanes (POSS). We described herein their associations with Au-NPs, with a main part devoted to fullerene clusters.

#### 4.1.1. Fullerene (C_60_) Clusters

**Scheme 7:**
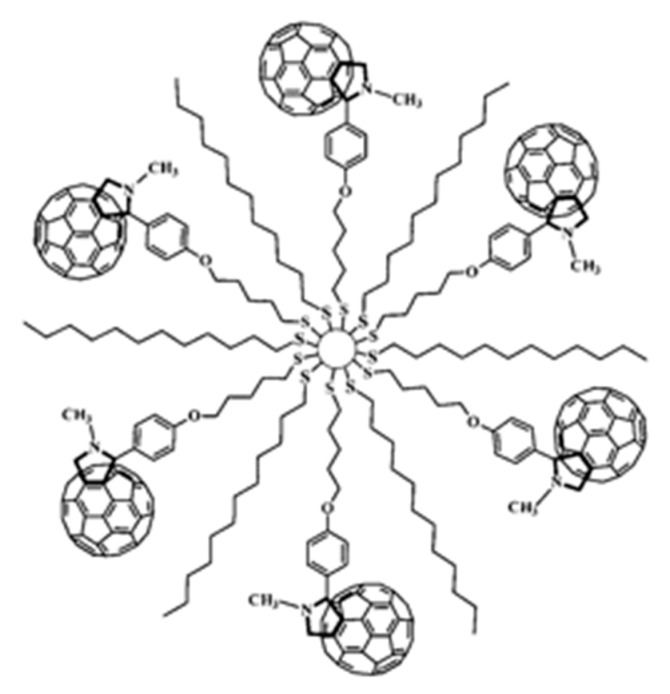
Scheme of one example of fullerenethiol-functionalized gold nanoparticle. Reproduced with permission from [69], ACS, 2002.

Fullerenes have been extensively studied since the discovery in 1985 of a new allotropic form of elemental carbon: C_60_ [64]. Due to their electronic, spectroscopic, and structural properties as well as their controllable functionalizations, C_60_ has been, among all existing fullerenes, the most widely used for the design of composite materials [65]. The first report concerning the association of C_60_ with Au-NPs was published to the best of our knowledge in 1998 by Mathias Brust [66]. In this work, C_60_ was used to mediate the aggregation of free gold nanoparticles in toluene. Then, progress in the functionalization of C_60_ and the possibility to introduce only one covalent function onto C_60_ led to Au-NPs functionalized by fullerenes. In 2001, H. Fujihara et al. described the first thiolated fullerene-functionalized Au-NPs. In this nanocomposite, Au-NPs were co-stabilized by fullerene-thiol and octanethiol (See Scheme 7) [67,68]. A similar approach was developed by K.G. Thomas et al. in 2002 by use of an alkyl chain between C_60_ and Au-NPs (Scheme 7) [69], contrary to Y.-S. Shon et al. who used an aromatic aminomercaptophenol as a linker [70]. However, with Fujihara, fullerene-thiols were grafted on the particles with other alkanethiols as co-stabilizing agents (C_8_H_17_SH or C_12_H_25_SH). Introduction of the fullerene-thiol moiety occurred via a ligand exchange process except for Shon, who tried the direct method by use of a mixture of C_60_-Ph-SH and C_8_H_17_SH. All these nanocomposites (Au-S-R-C_60_) were prepared for electrochemical or photoelectrochemical applications.

**Scheme 8:**
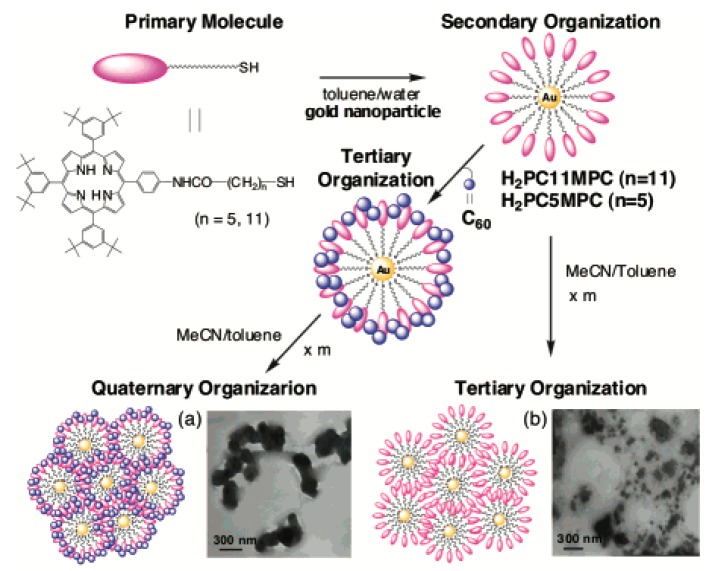
Scheme of nanocomposites based on the association of porphyrins and C_60_ onto Au-NPs surface. Reproduced with permission from [72], ACS 2003.

Other developments involving C_60_ and Au-NPs concern their combinations with γ-cyclodextrines [71] to form network aggregates or with porphyrins to design photovoltaic solar cells (See Scheme 8) [72]. Recently, Au-NPs stabilized with fullerene—offering multiple binding modes [73] or functionalized with fulleropyrrolidine [74]—were reported.

#### 4.1.2. Carborane Clusters

**Figure 1:**
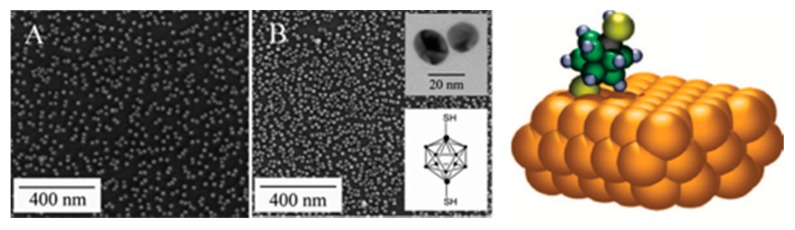
SE TEM images (**A**,**B**) of Au-NPs functionalized by the carboranethiol 1,12-(HS)2-1,12-C2B10H10 and the side view of space-filling models of same carboranethiol drawing in the capture on a gold (111) surface. Reproduced with permission from [82], ACS 2008.

Polyhedral carborane clusters have been extensively studied due to their potential applications in areas such as medicine, catalysis, and materials science [75,76]. Intense efforts have also been devoted to reach a controlled functionalization [77,78]. In particular, this cluster can be directly functionalized with one- or two-thiol functions through -B-SH bonding [79,80]. To date, only two articles describe the direct functionalization of Au-NPs by carboranethiol (See Figure 1). Both studies were done by Baše et al., in which they studied the nature of the interaction between these carboranethiol clusters and Au-NPs. Electrochemical properties of these nanocomposites were also investigated [81,82].

The potential applications of carborane-based Au-NPs is extremely broad since examination of ion transport across biological membranes [83] or cancer applications [84] were notably developed.

#### 4.1.3. POSS Clusters

**Scheme 9:**
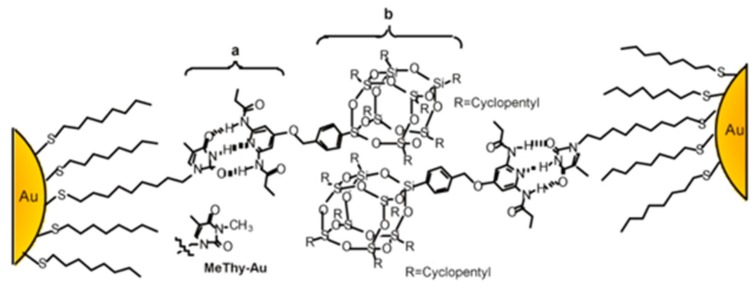
Scheme illustrating POSS clusters (**b**) in interaction with Au°-nanoparticles, functionalized by thymine through three-point hydrogen bonding recognition (**a**). Reproduced with permission from [86], RSC, 2002.

Polyhedral oligomeric silsesquioxanes (POSS) are clusters that have been intensively employed in materials sciences, particularly as mineral charge. They are considered nano-building blocks due to their diameters (1.5 nm), and are easily functionalized by one or several organic groups [45]. In fact, POSS were used for the first time as functionalizing agents by G. Schmid et al., who successfully introduced alkylthiol groups on the clusters. Another feature of that study was related to the nature of the nanoparticles which were Au_55_Cl_6_ clusters [85]. Another strategy was used by Rotello et al. who used electrostatic or hydrogen bonding interactions to link POSS to Au-NPs in order to obtain self-assemblies. For hydrogen bonding interaction, the approach consisted of the functionalization of Au-NPs by thymine functions (Thy-Au) and in the monofunctionalization of POSS by a diaminopyridine group (POSS-DAP) (See Scheme 9). Both functions being complementary, the recognition process of POSS towards Au-Thy resulted in the generation of nanocomposites exhibiting spherical aggregation resulting from the crystalline packing of non-polar POSS-DAP [86]. The second study consisted to devise POSS with eight ammonium groups (octa-ammonium POSS^®^, OA-POSS), and to generate self-assemblies with carboxylate capped Au-NPs. Syntheses of these aggregates were performed, in both studies, with two sizes of Au-NPs [87].

Over the years, potential applications of POSS-based Au-NPs broadened, extending from colorimetric detection [88] to reduction reactions [89].

### 4.2. Silica Coating

**Scheme 10:**
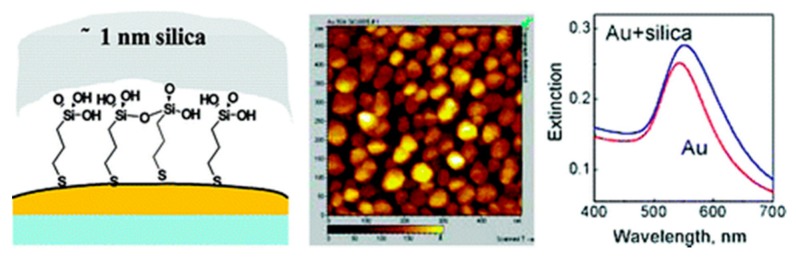
Scheme showing the method to coat Au°-NP by SiO_2_ through a mercaptosilane group, with TEM image and UV-Vis figure of these Au°*_core_*/SiO_2*shell*_, reproduced with permission from [96], ACS 2007.

During the design of a new nanocomposite, whatever its nature, two parameters are fundamental: colloidal stability of the resulting solution and ease of functionalization. Another important point concerns the possible desorption of the ligands from Au-NPs. In this field, silica appears an excellent candidate to prevent that problem and particles coalescence. Moreover, silica is chemically inert, optically transparent, and easily functionalizable. In this context, silica-Au NPs have been developed for numerous applications [90]. Due to these different properties, numerous efforts were devoted to coat Au-NPs with silica and to control the thickness of the coating and on different form of NPs [91]. Mulvaney and Liz-Marzán thus developed an efficient way to prepare Au°*_core_*/SiO_2*shell*_ nanocomposites of controllable thickness. The first step of the synthesis consisted in functionalizing citrate-capped Au-NPs with aminopropyl-trimethoxysilane. Amine functions ensuring a good interaction with Au° surface, a complete coverage of Au°-surface by alkoxysilane groups was obtained, followed by the condensation of siloxane groups. Finally, the thickness of the silica coating was controlled by the complementary addition of sodium silicate [92]. Since this initial report, many studies were devoted to find alternatives to this synthetic procedure [93,94], to control the optical properties of the nanocomposites [95,96] or to functionalize silica shell by polymers [97] or by chromophores to get fluorescence enhancement (See Scheme 10) [98].

**Scheme 11:**
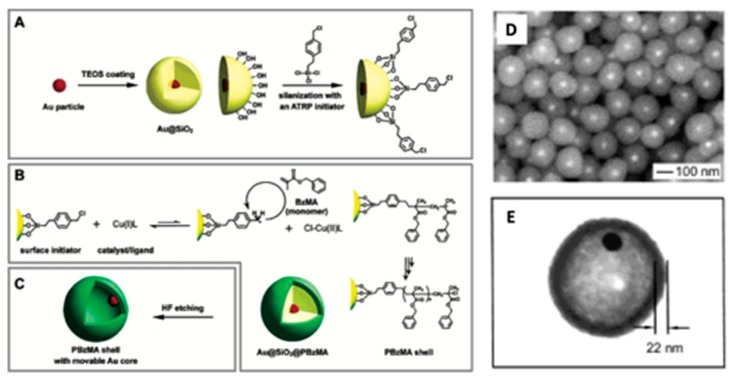
Schematic procedure used by Xia et al. to device PBzMA hollow beads containing movable gold cores (**C**), from Au@SiO_2_@PBzMA NP that realized from routes (**A**,**B**). The representative SEM before HF etching (**D**) and TEM images of Au@SiO_2_@PBzMA particles (**E**) after HF etching. Reproduced with permission from [99], ACS 2003.

An original device was reported by Xia et al. in 2003 [99]. Their device consisted to first perform an Au°*_core_*/SiO_2*shell*_ and then to functionalize its surface with a uniform polymer as a second shell (poly (benzyl methacrylate)). By treatment with an aqueous solution of HF, the SiO_2_ shell dissolved, leading to a hollow bead with movable gold cores (See Scheme 11).

### 4.3. Combination Au-NPs and Carbon Nanotubes.

**Scheme 12:**
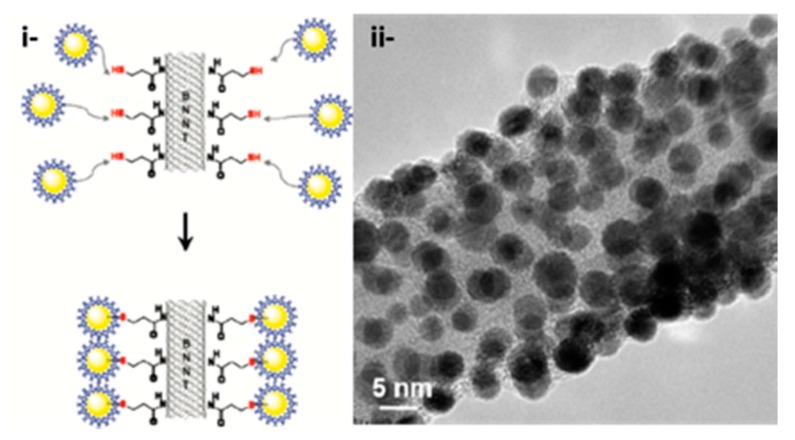
(**i**) Schematic representation of decorative boron nitride nanotubes, functionalized by thiol pendant groups, by Au-NPs, i- route of synthesis and (**ii**) corresponding TEM image, reproduced with permission from [105], ACS 2004.

As for fullerenes, carbon nanotubes, which were discovered in 1991 [100], have largely been studied for their unique structural, electrical, and mechanical properties, paving the way towards numerous practical applications [101]. In the specific case of these nanocomposites—and due to the difference of size—it is more obvious that it is the nanotube (NT) that is functionalized by Au-NPs. However, these composites are very promising materials for various applications, ranging from optic to electronic, biosensors or catalysis. [101,102]. Different strategies were investigated to decorate nanotubes by Au-NPs. One of the first methods consisted to reduce a Au^III^ salt onto the surface of carbon nanotubes by thermal decomposition [103]. However, the main developments concerned the electrostatic and covalent anchorage of gold nanoparticles on nanotubes. In all these approaches, a pre-functionalization of the NT surface by oxidative treatment with HNO_3_ or H_2_SO_4_-HNO_3_ was required, leading to the formation of carboxylic acid groups [104]. Then, conventional organic reactions could be performed onto NT’s surface due to the presence of these carboxylic groups or other functional groups. Another similar approach consisted to introduce amino groups on NT surface with boron nitride nanotubes (See Scheme 12) [105]. Another aspect concerning the presence of carboxylic acid groups is the modification of the electrostatic charge of the nanotube. Thus, the anionic character of NTs allowed the adsorption of cationic polyelectrolyte chains and subsequently the interaction with negatively charged Au-NPs [106,107].

**Scheme 13:**
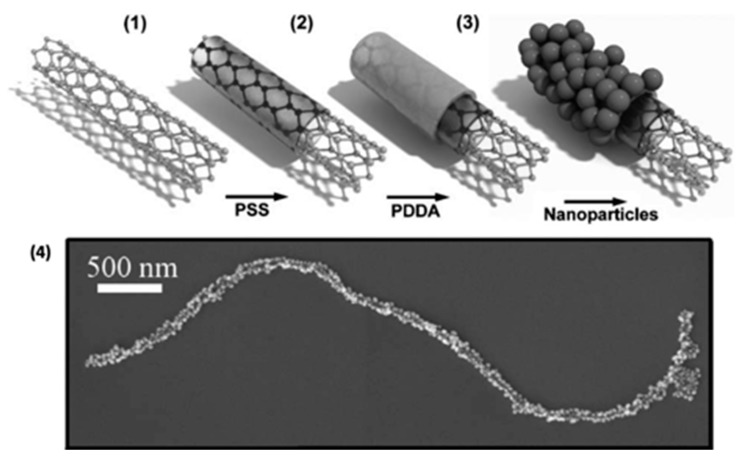
Schematic illustration of a non-covalent functionalization of CNTs comprising (**1**) polymer wrapping using poly(sodium 4-styrenesulfonate) (PSS), (**2**) self-assembly of poly- (diallyldimethylammonium chloride) (PDDA), (**3**) nanoparticle deposition and (**4**) SEM image (top) of one monolayer of Au@SiO_2_ nanoparticles assembled onto a carbon nanotube, reproduced from [108], RSC 2006.

Another approach consisted in introducing a silica coating onto NTs surface via thiol or amino functions [108]. This silica coating can be functionalized in a second step, as reported by Bottini et al. (See Scheme 13) [109,110].

An alternative and elegant approach consisted to used π–π stacking interactions of aromatic molecules such as pyrene to functionalize NTs. This strategy was used by Huang et al. with a 1-pyrene-methylamine as linker between NTs and Au-NPs [111].

## 5. Functionalization by Polyoxometalate Compounds (POM)

Smaller than gold nanoparticles but lying between the colloidal and the molecular range, the polyoxometalates (POM) species based on Mo or W constitute a full class of nanobuilding blocks. These metallic oxo-clusters play a great role in many areas. Their applications are due to the combination of several value-adding properties, and to their ability to behave as fully oxidized/(photo)-reducible compounds [112].

Due to their anionic charge, main links between POMs and metallic nanoparticles are conducted by electrostatic interactions. In this case, POMs play protecting-ligand shell role surround metallic nanoparticles [113,114,115,116]. The development of organic-inorganic hybrids derivatives POMs allowed another class of covalent POMs surrounding metallic nanoparticles. These organic-inorganic hybrids derivatives POMs are designed from a lacunar POM, containing a surface of more nucleophilic oxides, on which organosilyl groups (RSi (OR)_3_ type), can ensure a covalent link W-O-Si [117]. Mayer et al. [118] used this hybrid POM, to introduce thiol groups. Using this strategy, gold nanoparticles are covalently surrounded by hybrid POM. And the mercaptoorganosilyl group ensures the link between nanoparticles and POMs [117]. Other similar developments have been later reported by Shweta et al. [119].

**Scheme 14:**
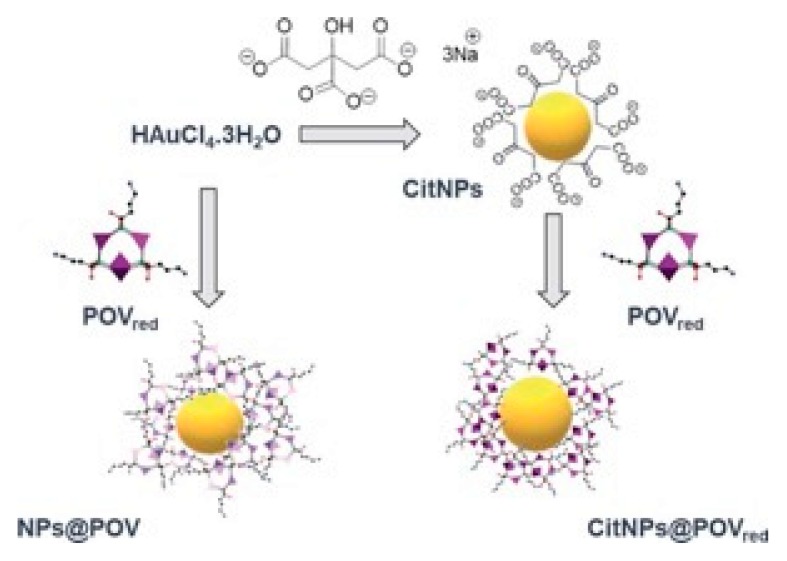
Schematic representation of CitNPs, CitNPs@POVred and NPs@POV synthesis, reproduced with permission from [121], RSC 2019.

Different strategies have also been used to modify POMs such as the functionalization of the POM core by organo-amino groups, reported in 2019 by the Leroy’s Group [120]. In this case, POMs could be used as a reducing and coating agent to design these gold nanocomposites. Another route consists in using a reduced polyoxovanadate functionalized with bisphosphonate molecules in order to prepare in a single step hybrid organic–inorganic polyoxometalate decorated gold nanoparticles. These new nanocomposites were shown to strongly inhibit P. aeruginosa and S. epidermidis biofilm growth (See Scheme 14) [121].

## 6. Functionalization by Organometallic Complexes

Functionalization of Au-NPs by organometallic (OM) complexes is predominantly trusted by ferrocene complexes due to their electronic properties valuated mainly in redox based sensors. But other OM based on ruthenium or rhodium as metals have also been grafted onto Au-NPs for catalysis applications as recently summarized by Wilton-Ely [122]. In this part, we will review the different OM complexes and extend our overview to metallodendritic complexes.

### 6.1. Single Ferroncenyl Complexes

**Scheme 15:**
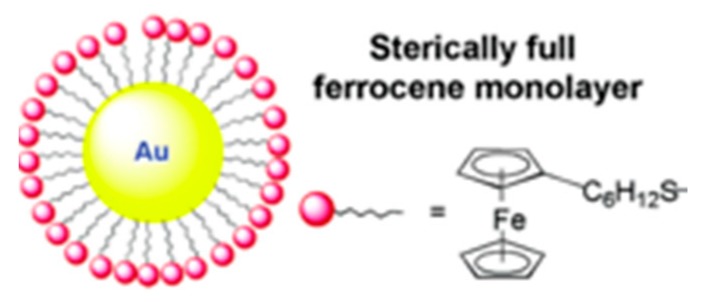
Scheme of a typical ferrocenethiophenol, figure reproduced with permission from [124] ACS 2007.

Brust’s method has considerably contributed to gold chemistry and particularly to the functionalization of Au-NPs by various groups. Notably, this method enabled the development of ferrocenated NPs, by use of ferrocene substituted thiols acting as functionalizing agents. For the direct synthesis of Au-NPs, various linkers have been tested, such as ferrocenylhexanethiol (See Scheme 15) [123]. This molecule exhibited a linker of sufficient length to induce a good control of the size monodispersity of NPs [124]. Linkers containing aromatic groups have also been used such as the ferrocenethiophenol group [125,126,127].

The other strategy to functionalize Au-NPs consists in the ligand place-exchange reaction. Murray and co-workers were one of the first research groups to use this procedure for the heterofunctionalization of Au-NPs with various functional groups. In a first study, ferrocenyloctanethiol was thus exchanged with different alkanethiols [128]. In a second, the same group devised mono-functionalized ferrocenated Au-NPs by use of ferrocenyl octanethiol [129]. An alternative to this procedure was recently developed by Astruc et al., consisting in the functionalization of Au-NPs by cross olefin metathesis. In that recent procedure, Au-NPs are prefunctionalized by alkene-terminated groups (methyl acrylate). Then, the cross-metathesis of a ferrocenylmethyl acrylate and alkene-substituted Au-NPs using Grubbs’ catalyst, gave the nanocomposite [130].

**Scheme 16:**
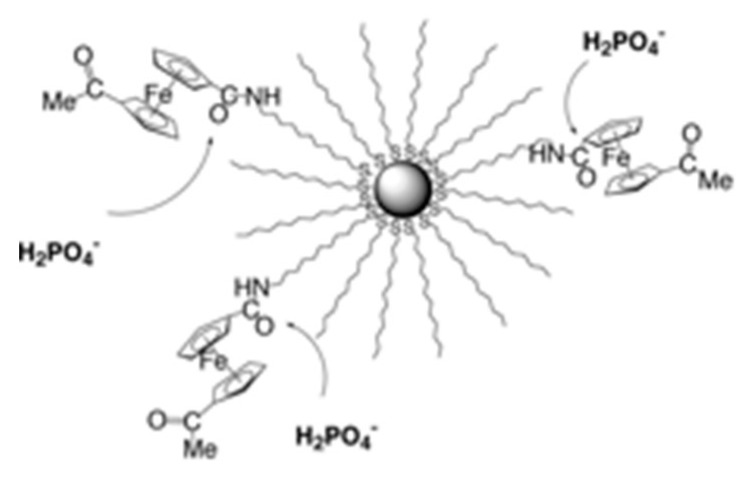
Scheme of amidoferrocenated-Au NPs and the recognition of H_2_PO_4_^−^ anions, reproduced with permission from [133] ACS 2002.

Most of the ferrocenated Au-NPs were designed for electrochemical applications. However, parallel to this first research topic, ferrocenated Au-NPs were also prepared for redox sensors applications towards H_2_PO_4_^−^ and HSO_4_^−^ anions. One of the first researchers to develop this application was Astruc and co-workers, in 2000, who used amidoferrocenyldodecanethiol groups [131]. They notably designed amidoferrocenated groups to check the recognition properties of these different ferrocenated Au-NPs [132]. The recognition process was based on a double hydrogen-bonding interaction between the amido group of the amidoferrocenium and the anion. In Scheme 16, the ferrocenated NPs used in this study is presented [133].

To improve the redox properties or the sensitivity in anions sensing, different poly-ferrocene complexes have been proposed. The simplest one was biferrocene. Nishirada et al. synthesized biferrocene-terminated alkanethiol to functionalize Au-NPs and analyzed their electrodeposition [134,135,136]. Another study combined ferrocenyl and biferrocenyl groups with terpyridine ligands to generate a biferrocenyl-functionalized ruthenium (II) complex-Au-NPs for redox applications [137]. Then, to perform the recognition of H_2_PO_4_^−^ anion, Astruc et al. synthesized a dendron bearing three amidoferrocenyl, or three silylferrocenyl groups. These three ferrocenyl dendrons were introduced onto Au-NPs surface by a ligand place-exchange process [138]. In particular, they extended this approach to a larger metallodendron containing up to nine ferrocenyl units. In this way, they designed ferrocenyl-dendron-Au-NPs exhibiting 360 ferrocenyl units (for the largest dendron) at the periphery of the nanocomposites. With these nanocomposites, various anions could be recognized, such as the well-known Adenosine-5′-triphosphate [139,140]. Recycling of organic compounds is an active research field and ferrocenyl-based Au-NPs have been notably used as catalysts for recycling 4-nitrophenol. [141]. But recent advances on surface functionalization has recently been obtained with ferrocenyl-Au NPs for which a deformation of the organic coating was observed. As a result, a stable layer or ferrocenyl-Au NPs adsorbed at the metal surface could be obtained [142].

### 6.2. Variety of Organometallics-Au-NPs.

**Scheme 17:**
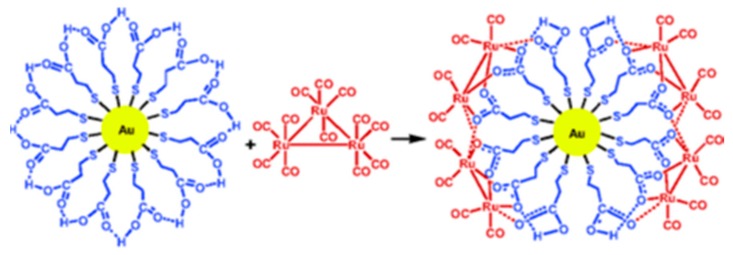
Route of functionalization of Au-NPs by trinuclear ruthenium complexes, reproduced with permission from [145] ACS 2006.

Examples of Au-NPs functionalized by organometallic complexes other than ferrocene are relatively scarce. In this field, the first example of this category is a tetranuclear iron complex. Relatively close to ferrocene in structure, this organometallic complex [Fe(η^5^-C_5_H_5_)_3_(µ_3_-CO)_4_(η^5^-C_5_H_4_CONH(CH_2_)_11_SH] was used as redox sensors for two typical phosphate anions (ATP and H_2_PO_4_^−^). Palladium (II) OM complexes have large catalytic properties. Fratoddi et al. recently synthesized an OM Pd (II) thiol complex in which the thiol function was directly linked to the Pd (II) center. This nanocomposite was prepared via the direct functionalization process (Brust’s method) [143]. For applications in catalysis, some OMs based on Ru (III or II) or Rh (I) as metals were synthesized [144]. Dinuclear ruthenium complexes were prepared with two or four alkylthiol pendant groups to ensure a strong grafting of OM onto Au-NPs surface [145,146]. The rhodium OM complex was mononuclear and was linked to Au-NPs by an amidododecanethiol group (See Scheme 17) [145].

## 7. Functionalization by Coordination Complexes from D-Block Elements

### 7.1. Prussian Blue Derivatives

**Scheme 18:**
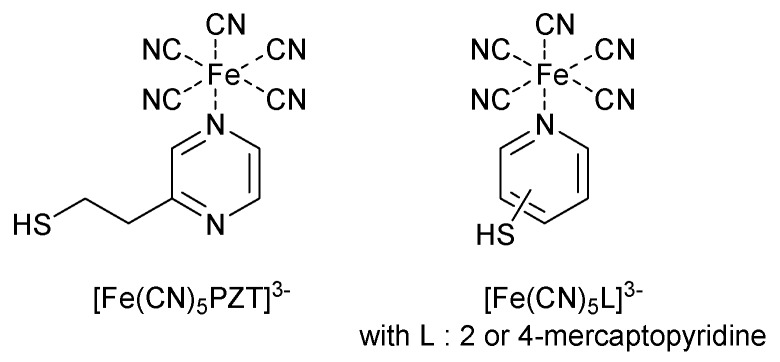
Prussian blue-modified complexes.

Several nanocomposites resulting from the association of gold nanoparticles with Prussian blue derivatives have been reported in the literature. Various strategies have been developed to generate the nanocomposite. The first is based on a one-step procedure combining an electrochemically controlled generation of gold nanoparticles concomitant with the electrochemical formation of Prussian blue (PB) [147]. Such a modified electrode has been successfully used for catalyzing the reduction of hydrogen peroxide and for the amperometric detection of H_2_O_2_ with a nanomolar sensitivity. PB@Au nanoparticles with a diameter ranging from 20 to 50 nm were thus obtained (See Scheme 18). Previously, the same type of functionalization had been performed with gold particles stabilized with dendrimers (PAMAM: polyamidoamine) by potential cycling electrodeposition [148]. In that case, particles were smaller (3 nm) and exhibited a narrower size distribution. PB@Au nanocomposites can also be prepared by a chemical procedure [149]. Hence, reduction of ferric ion in water in the presence of Fe(CN)_6_ and preformed gold particles resulted in PB-functionalized particles with an average size of 50 nm. Then formation of PB@Au-multilayer thin films by the Langmuir–Blodgett technique resulted in a hydrogen peroxide sensor. In both cases (chemical or electrochemical method), functionalization of the particles resulted from an electrostatic interaction between PB and gold particles.

Grafting of metal complexes on Au particles can also be realized using linkers such as 2-pyrazin-2-ylethanethiol [150], or 2- and 4-mercaptopyridine [151]. In the last case, aggregation of the particles was observed by use of the 2-mercaptomyridine, whereas stable particles were obtained with 4-mercaptopyridine.

### 7.2. Terpyridinyl Metallic Complexes

**Scheme 19:**
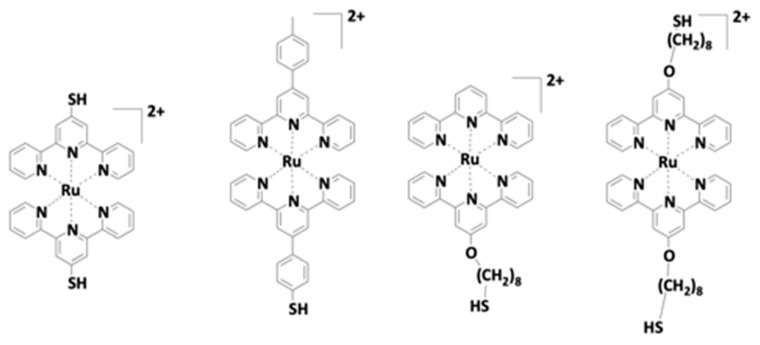
Some examples of *bis*-terpyridine Ru (II) complexes, with direct link between Au-NPs and metallic complex or through an alkyl chain.

Complexed and none-complexed terpyridines have also been used to prepare stable nanocomposites. As a first example, a thiol pendant group was used to anchor the terpyridine [152]. In that work, stable particles were obtained at low concentration of terpyridine by ligand exchange on weakly stabilized particles. Increase of the concentration resulted of a none-negligible interaction of the terpyridine moieties with the surface, aggregating the particles (See Scheme 19).

**Scheme 20:**
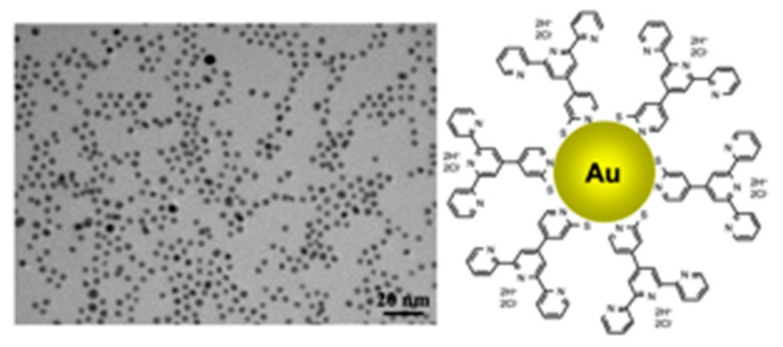
Au-NPs stabilized by terpyridinium ligands for a post-complexation, reproduced with permission from [158] RSC 2006.

The same reaction, done in the presence of an excess of zinc triflate, afforded in all cases stable particles, with stabilization of the particles being ensured by electrostatic repulsions generated by complexes. The dramatic influence of the counterion on the stabilization process was also evidenced. A thiol group can also be introduced on the terpyridine core by use of aliphatic spacers of different length [153]. After anchorage of the terpyridine on the particles, and addition of different cations, such as Fe(II), Zn(II), Cu(I), or Ag(I), self-assembly of the particles was observed, resulting from the coordination process between terpyridines linked to separated particles. During that work, Rotello et al. controlled interparticle spacing by modification of the length of the spacer and the stability of the aggregates by changing the cation. The same strategy was developed by another group to prepare different superstructures by use of copolymers as template [154]. The size of the aggregates can also be controlled by the nature of the cation [155]. By use of alkali cations, a weak coordination of the cation was observed, resulting in large 3D assembly. After dissociation of these aggregates with DMF, which gave individual gold particles—and addition of Co (II) cation, inducing a strong coordination with the terpyridine—a highly dispersed 3D spherical assembly was obtained. It was the first example of transformation of a 3D nanonetwork. More recently, a 2D network was prepared according to a novel procedure [156]. Thiol-substituted terpyridine were first deposited as a monolayer onto the oxide surface of a Si/SiO_2_ substrate by the Langmuir-Blodgett method. Then, addition of Fe (II) led to the formation of the complex with terpyridine grafted on adjacent particles. The influence of the conjugated linkers on the conductivity of the network was investigated. Metal complexes can also be generated to stabilize particles without creating inter-particle interactions. Hence, reaction of the grafted terpyridine with an excess of Ru(terpyridine)Cl_3_ gave the nanocomposite [157]. Then, a partial ligand-exchange reaction on the former nanocomposite with thio-substituted pyrrole led, after electropolymerization, to the first example of a polypyrrole nanocomposite containing metal complexes and metal nanoparticles. A similar functionalization of particles was performed with a rigid fully conjugated linker between the metal complex and the particles (See Scheme 20) [158]. Communication between particles and complexes was electrochemically evidenced by modification of the oxidation potential of Ru (II) in the metal complex. As final nanocomposite, a preformed Ru (II) terpyridine complex was attached to gold nanoparticles by ligand exchange to prepare a voltage-driven molecular switch [159]. This Ru (II) complex was also associated to a second metal complex (ferrocene) and the mutual influence of both metal complexes grafted on particles was investigated by electrochemistry [159].

### 7.3. Polypyridyls Metallic Complexes

**Scheme 21:**
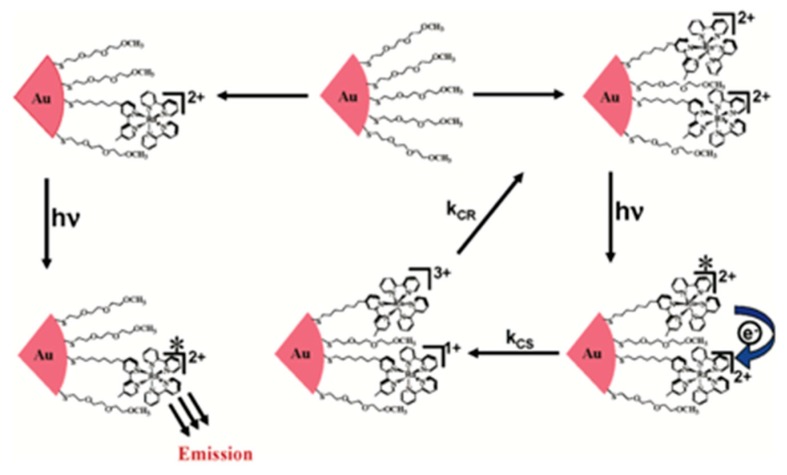
Au-NPs stabilized by *tris*(bipyridine) Ru (II) complexes for a light-induced processes inside these Au°-(S-C7-Ru), reproduced with permission from [162], ACS 2006.

Polypyridyl metal complexes based on bidentate ligands have been extensively used as IME for Au nanoparticles. They can be immobilized onto Au-NPs either by electrostatic interaction or by use of an anchoring group [160,161]. Grafting of electrochemical or optical probes onto the surface of gold nanoparticles is another active field of research. Among all existing metal complexes combining electrochemical and luminescence properties, the well-known *tris*(bipyridine)ruthenium (II) is a complex of choice. Functionalization of gold nanoparticles by bipyridine complexes can be realized according to two different strategies. In the first, metal complexes directly interact with the particles via electrostatic interactions. Several reports concern the direct grafting of the unmodified *tris*(bipyridine)ruthenium (II) complex, its intrinsic properties making it a convenient probe to evaluate the possible energy transfer, electron transfer or enhanced intersystem crossing rate constant between the gold nanoparticles and the complex. Particularly, a luminescence-quenching of Ru(bipy)_3_^2+^ onto gold nanoparticles was reported by Murray et al. [162], metal surfaces being known to efficiently quench molecular excited states (See Scheme 21).

Quenching of fluorescence has been studied with tiopronin-protected gold nanoparticles of different diameters. A clear increase in quenching efficiency with the diameter core was evidenced, a successful quantification of this phenomenon being possible, thanks to a reversible electrostatic binding between the particles and the fluorophore. By addition or not of KCl electrolyte in the solution, electrostatic interactions between NPs and the complex were modified, with the electrolyte hiding the carboxylate binding sites of the tiopronin. Another study was devoted to quantifying the number of adsorbed complexes onto the gold surface of two differently stabilized particles using time-correlated single-photon counting spectroscopy [163]. The kinetics of adsorption of the complex, as well as the size and temperature dependence of Au-NPs, were other parameters investigated for the comprehension of the luminescence-quenching [164]. Other fundamental questions were studied such as chromophores density, size, or temperature dependence of optical and surface properties for immobilized Ru(bipy)_3_^2+^ complex on Au-NPs or nanorods via Au-S bonding [165,166]. All former studies were realized with the aim to find an efficient control of the energetic or the electronic communication between the particles and the complex for future biological, catalytic, optical, or electronic applications.

Ru(bpy)_3_^2+^ complex is also widely studied for its electrochemiluminescence (ECL) properties. Using this property, various modified electrodes were prepared as for example for solid-state ECL detection in capillary electrophoresis [167]. In order to optimize the ECL detection of the indium tin oxide (ITO) electrode, Ru(bpy)_3_^2+^-AuNPs aggregates were prepared and immobilized on the conductive support via Au-S binding.

These systems exhibited a significant improvement of ECL intensity with a detection 10^4^ times more sensitive than without grafted gold nanoparticles. Modified electrodes were also used for the selective detection of biochemical molecules such as pentoxyverine [168]. Others hybrid materials were prepared using polypyridinyl complexes derived from Ru(bpy)_3_^2+^ complex. As a first example, a complex bearing three thiol pendant groups was used to fabricate an ITO electrode with self-assembled layers of ruthenium complexes [169]. Formation of stable and well-ordered 3D structures at the electrode surface was thus observed and a noticeable increase of the photocurrent response with the number of layers was evidenced. Improvement of such 3D-assemblies could be valuable in ruthenium (II) dye-sensitized solar cells.

**Scheme 22:**
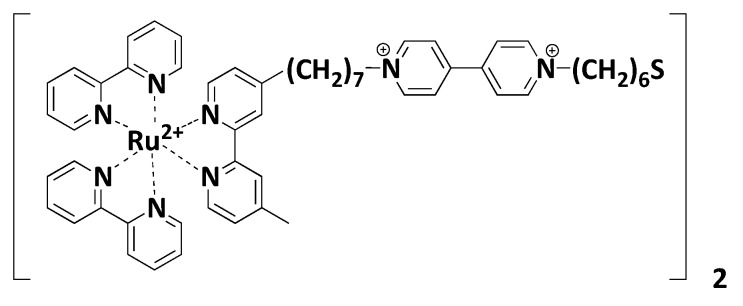
Example of Ru complex - viologen linked thiol to immobilize onto Au-NPs [170].

Modified electrodes can also be prepared using an electroactive spacer such as a viologen group] (See Scheme 22) [170]. By deposition of functionalized Au-NPs onto a gold electrode, a sensitivity more than 15 times larger for the electrode was observed for the anodic photocurrent detection. A participation of the viologen moiety by its one-electron reduction process was demonstrated. Continuation of that initial work consisted to study the influence of the nanoparticles size on the photocurrent response.

Nanostructured assemblies of particles with diameters ranging from 50 to 100 nm presented the optimal photocurrent efficiencies [171]. Based on the strategy that an increase of the ionic force is known to cause flocculation of Au-NPs, a novel method to elaborate these nanostructures was developed, still using these viologen-substituted ruthenium complex [172]. In that work, influence of the nature of the electrolyte, in particular of the anionic moiety was studied due to the fact that it can modify the morphology of these systems and subsequently alter the photocurrent responses. Preceding these viologen-based systems anchored to Au-NPs via a thiol group, several viologen systems based on electrostatic interactions between the Ru(bipy)_3_^2+^ complex and citrate-capped nanoparticles were investigated [173,174]. Photoelectrochemical cells of low efficiencies were obtained, resulting in an energy-transfer-quenching of the viologen sensitizer by the gold nanoparticles. Gold nanoparticle assemblies can also be easily obtained by exploiting the complexation of a metal cation by pyridine moieties [175]. Using this strategy, thin films were obtained and exhibited diode-like responses.

Always with a polypyridinic environment around the redox center, other anchoring groups have been used to functionalize and stabilize gold nanoparticles. In a recent work, a Ru(bipy)_3_^2+^ complex with a terminal amine group was used to rationalize the effect of the nanoparticles on the radiative and the none-radiative rates of a phosphorescent compound [176]. Phosphorescent molecules were grafted on the particles via a selective recognition technique: nanoparticles were first prepared in the presence of streptavidin with an excess of bovine serum albumin. Streptavidin being known to be a selective sensor for biotin, phosphorescent molecules were thus functionalized with biotin. Then, grafting of these molecules onto NPs was realized, based on the biotin-streptavidin recognition process. Another example, also based on the biotin-streptavidin recognition, was developed for the assembly of proteins and gold nanoparticles on DNA templates [177]. Other properties, such as the supramolecular control of valence-tautomeric equilibrium of a bistable cobalt complex was also studied [178]. Influence of the anchorage on thermodynamic parameters was demonstrated, caused by surface confinement resulting from the attachment of the valence tautomer.

**Scheme 23:**
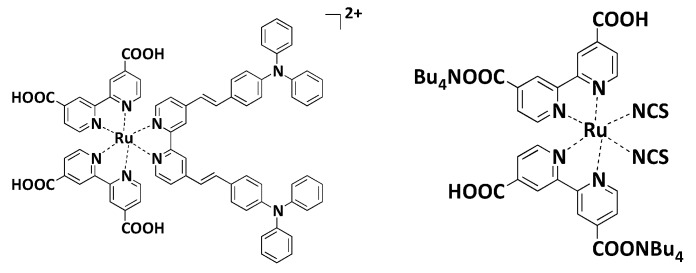
Ruthenium (II) bipyridyl complexes immobilized onto Au-NPs, through amino or isothiocyanate groups developed by Pérez Leon et al. [180].

Until now, only nanocomposites obtained by electrostatic interactions or by use of long aliphatic chains with thiol end groups have been presented. In these systems, no electronic communication was possible through the non-conjugated spacer. Recently, Mayer and co-workers reported the synthesis of several polypyridinic ruthenium complexes based on phenanthroline bidentate ligands with fully conjugated spacers, enabling a direct communication between the particles and the complex (See Scheme 23) [158,179]. Modification of the redox potential of the ruthenium complexes was observed, evidencing an electronic communication.

Bipyridyl complexes with conjugated linkers have also been investigated for surface-enhanced Raman scattering measurements [180]. In particular, the influence of the solvent was studied, and results demonstrated different ways of adsorption for the complex, with these phenomena depending on the solvents. The ruthenium complex used in this study was composed of a ruthenium center chelated by three bipyridine ligands modified either by the addition of a conjugated spacer of triphenylamine, acting as the pendant group, or by the addition carboxylate groups. In all former examples, only functionalizing agents with only one anchoring point have been presented. But they can also have two anchoring groups. This is the case for a complex prepared with a phenanthroline exhibiting two thiol groups [181].

### 7.4. Coordination Complexes Grafted on Au-NPs for Multiple Applications

**Scheme 24:**
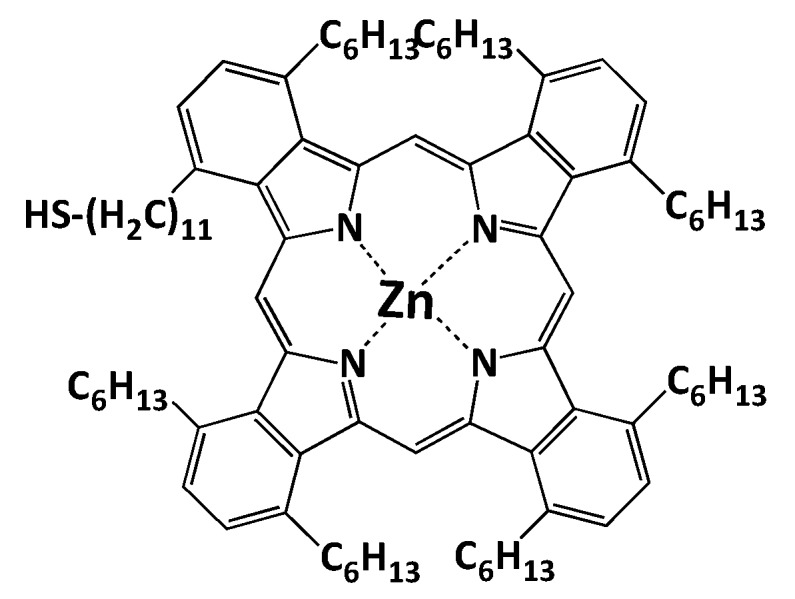
Zinc (II) phthalocyanine complex used to coat Au-NPs for photodynamic therapy [183].

Corresponding ruthenium complex was used to create nanocomposite junctions between electrodes. Nanocomposites were prepared via a sulfur-exchange reaction and grafted between two facing gold electrodes with a micrometric gap. Conductivity measurements were investigated and demonstrated the efficiency of such electric self-assemblies, with the presence of the ruthenium cation allowing an increased conductivity and a lower energy barrier. Concerning other practical applications, bidentate ligands such as bisoxazoline were used to prepare complexes valuable in catalysis. Hence, a chiral copper-bisoxazine complex covalently linked on gold colloids has been used for an enantioselective ene-reaction between 2-phenylpropene and ethylglyoxylate [182]. Use of this original homogeneous catalyst resulted in reactions with high yields and high enantiomeric excess. Another advantage of this catalyst is that it can be easily separated from the reaction mixture by filtration, and is thus reusable. Zinc (II) phthalocyanine was anchored to Au-NPs for another application (See Scheme 24) [183].

**Scheme 25:**
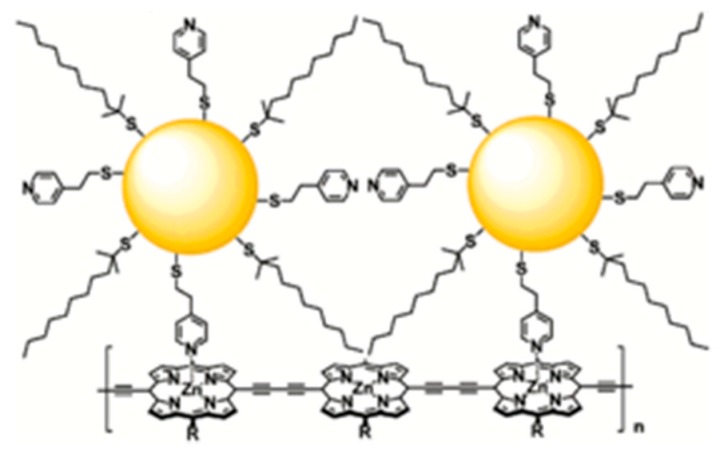
1D- Assembly of Au-NPs chemically linked to p-conjugated porphyrin, reproduced with permission from [185], ACS 2007.

By grafting the photosensitizer onto Au-NPs, the generation of singlet oxygen was obtained with an enhanced quantum yield, as compared to the free photosensitizer. Such systems could be potentially used for the delivery of photodynamic photosensitizer agents in photodynamic therapy. An interesting example of anion sensors based on porphyrins was recently reported in the literature [184]. The anchorage onto the NPs was realized in this case by use of four pendant groups derived from thiotic acid. The nanocomposite, when tested with six different anions, exhibited a significant increase in anion-binding affinities, when compared to the free metalloporphyrin. This improved affinity for anions resulted from the pre-organization of the porphyrins at the surface of the particles. Modified electrodes were also prepared with porphyrins. Based on a layer-by-layer deposition process of an electron acceptor, Au-NPs covalently linked to an ITO electrode could be prepared. Examination of these electrodes in photochemical experiments revealed the resulting photoelectrochemical cells to be of low efficiency with this photosensitizer [173]. In 2007, Ozawa and co-workers reported the preparation of porphyrin wires and the assembly of gold nanoparticles onto π-conjugated wires of porphyrins [185]. First, the polymer of porphyrins was deposited on a modified glass surface by the Langmuir-Blodgett technique. Then, the glass substrate was soaked with the particles capped with 4-pyridineethanethiol, resulting in the grafting of the particles on the glass substrate by interaction of the pyridine of the NPs with the porphyrin (See Scheme 25). The interest of that technique is that the grafting of Au-NPs on the glass substrate could only be observed where the polymer was previously deposited. As final systems based on porphyrins, biomolecules were grafted onto gold nanoparticles. Hemin chloride (Hem) and cytochrome c (Cyt c)—whose structures are derived from porphyrin—were immobilized on NPs and binding of a metabolic inhibitor (azide anion) was investigated [186]. Due to a reduced accessibility resulting of the grafting of the biomolecules, a thermal activation to bind the azide anion was necessary.

### 7.5. Carboxylates and Base Shiff Coordination Complexes

**Scheme 26:**
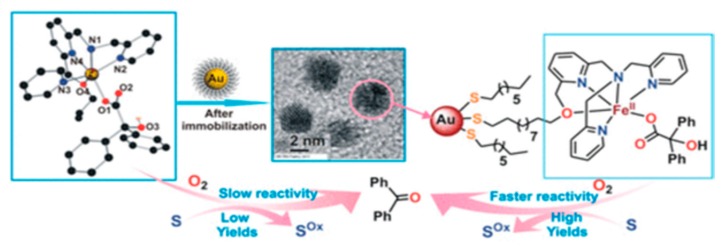
Illustration of the oxidizing ability of a dioxygen-activating from nonheme Iron(II)-Benzilate complex immobilized on Au-NPs, reproduced with permission from [187]. ACS 2019.

Complexes with various charged ligands have also been used to encase gold nanoparticles in monolayer organometallic metal-complex shells [187]. Hence, chelation of metal ions can be realized by carboxylate groups. Interest in such complexes relies in the weakness of the coordination which can be destroyed “on demand”, simply by using a stronger chelator that will dissociate the complex. The main applications of such systems are the detection of heavy metal ions [188,189], based on the aggregation of the nanoparticles in the presence of cations, the preparation of self-assembled monolayer [190,191,192,193], or nanocomposites further valuable in catalytic systems [194]. For the first two applications, nanoparticles were conveniently functionalized by mercapto-alkyl acid, the carboxylate group acting as the pendant group able to generate interparticle interactions under chelation with a metal ion (See Scheme 26).

**Scheme 27:**
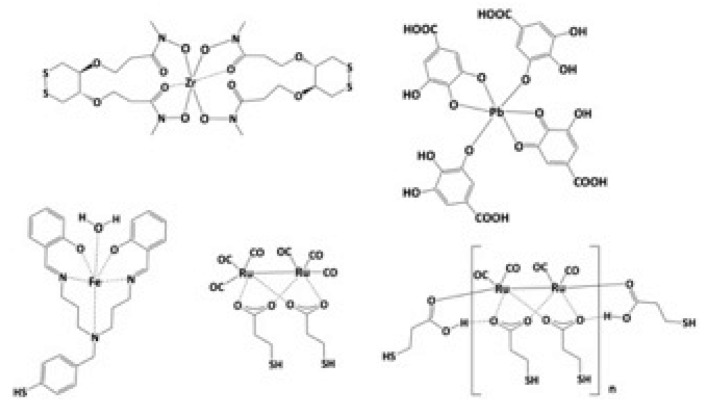
Examples of carboxylate, amidate, and phenolate based complexes used to coat Au-NPs.

For the last application, a multistep synthesis was developed to graft Ru-dodecacarbonyl complex, the carboxylic group known to react with the complex to form Ru-carbonyl-carboxylate complexes (See Scheme 27). By this strategy, a dimer or an oligomer was anchored to the particles. Other functions can be used to graft metal complex on particles. Hence, a *bis*-hydroxamate ligand has been used to generate coordination-based nanoparticle monolayers or multilayers with Zr^4+^ [195]. By use of a bifunctional molecule (gallic acid) bearing a carboxylate group as the capping agent and a hydroxy group to coordinate Pb (II) cation, a naked-eye detector was prepared. Due to the unique coordination behavior of Pb (II) cation, whose coordination number can be extended until 12, aggregates were obtained with Pb(II) cation.

On the contrary, addition of other metal cations leaves the nanoparticles isolated, as a consequence of the coordination of these cations with less ligands and the ability of Pb (II) cation to overcome interparticle electrostatic repulsions. Schiff base ligands have also been used to coordinate iron (III) cations. In that work, two complementary strategies have been developed to stabilize gold nanoparticles. The first work is based on the use of a neutral complex stabilized the NPs by steric repulsion (alkyl chain). The second approach is based on the use of a charged complex. Stabilization of the particles is done by electrostatic repulsion [196].

### 7.6. Bioinorganic Complexes

**Scheme 28:**
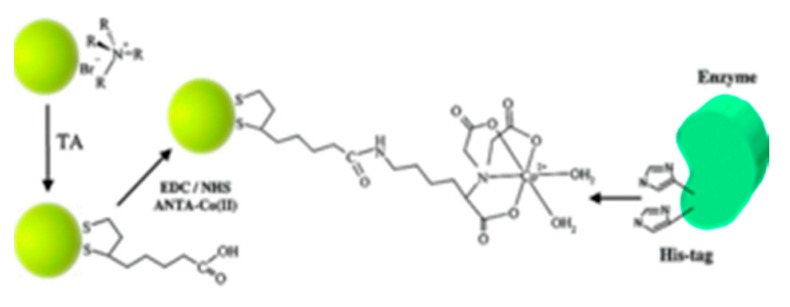
Biomolecules grafted on gold nanoparticles by immobilization of fully functional proteins onto the surface of Au-NPs through a thioctic acid function, reproduced with permission from [199]. ACS 2005.

The final type of ligands able to coordinate metal ions are all ligands based on biomolecules. A growing interest concerns the elaboration of nanocomposites modified with biomolecules due to their potential use in electronic, optical, and biosensor applications [136,197,198]. Proteins can thus be covalently attached to gold nanoparticles by generation of a Co (II) complex. The protein, obtained by genetic engineering, bears a histidine tag that can coordinate Co (II) ions by ligand exchange on a grafted Co (II) complex (See Scheme 28) [199]. Several examples of glyconanoparticles have been prepared to study carbohydrate interactions with Ca (II) cation in water [200,201]. Complexation of Ca (II) ions resulted in aggregation of the particles due to calcium cation-carbohydrate-carbohydrate interactions. These interactions are reversible, and addition of a strong chelator such as EDTA can redisperse the particles. Influence of the length of the spacer on complexation was also studied and the lowest detection level was obtained with the shortest linker.

**Scheme 29:**
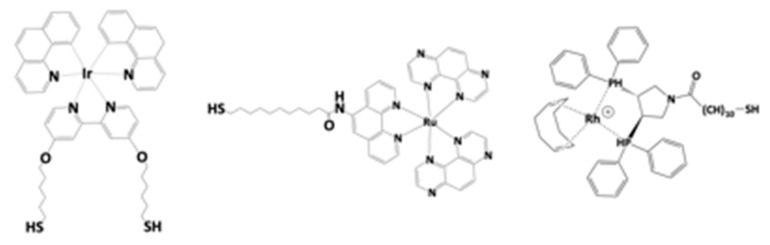
Catalysts or metallic complex for cell luminescence imaging used to surround gold nanoparticles.

Peptide-functionalized gold nanoparticles have also been prepared for Hg (II) ion detection [202]. Gold particles were first functionalized by a peptide exhibiting at both ends an amino and a carboxylic group. Anchorage of the peptide was done by the amino group. Due to the strong affinity of Hg (II) cations for amino groups, addition of the cation resulted in the detachment of the peptide from the surface of the particle. A 1D-linear assembly of colloidal particles was then observed. Addition of an alkali solution of EDTA decomplexed the peptide from the Hg (II) ion and the free peptide can absorb on the gold surface. DNA-functionalized gold nanoparticles have also been used for the colorimetric detection of various cations such as Hg (II) [203], Pb (II) [204,205,206,207,208], Cu (II) [209]. A ruthenium complex has also been used to graft DNA to gold nanoparticles, the ruthenium complex acting as the DNA-intercalating unit and the linker with the gold nanoparticles [177]. Anchorage of the DNA template containing 80 base pairs was done according to an original strategy. As the first step, streptavidin-coated gold nanoparticles were prepared. Then, the ruthenium complex was designed in such a way that the biotin-phenanthroline ligand was able to bind streptavidin, and the phenazine ligand was able to intercalate into the DNA duplex (See Scheme 29).

Several metal complexes have been covalently linked to gold nanoparticles, exclusively for catalytic and structural studies. Hence, chiral rhodium-diphosphine complexes on gold colloids have been used for enantioselective hydrogenation of α-acetamidocinnamate [146], and Ti-binolate complexes on particles have been synthetized and applied to the catalytic asymmetric alkylation of benzaldehyde [210]. A dimer of ruthenium complex has also been grafted on particles as a catalyst for ring-opening metathesis polymerization [144]. Other Ruthenium complexes have been grafted onto large nanoparticles for cell luminescence imaging, which revealed their biomolecular association with chromatin in the nucleus of cancer cells [211]. Recently, ruthenium complexes surrounding gold nanoparticles have been used to photocrosslink collagen [212].

### 7.7. Crown Ethers Devices

**Scheme 30:**
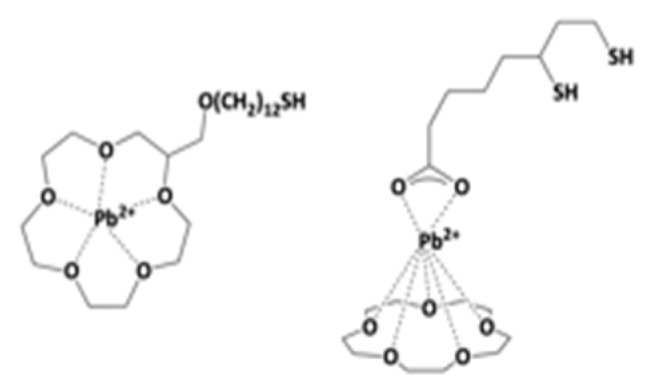
Two examples of crown ethers grafted onto Au-NPs.

Crown ethers were also used to chelate metal cations of the d-element. Two examples are reported in the literature. The first one concerns the covalent linkage of 2-(12-mercaptododecyloxy) methyl-15-crown-5 to gold nanoparticles, the final goal being the preparation of monolayer for the capture of Pb (II) cations [213]. A trapping capacity of 2.8 10^−10^ mol Pb (II) per cm² was obtained. The second example deals with bifunctionalized gold nanoparticles prepared for optical Pb (II) sensing [214,215]. Two different ligands (2-(12-mercaptododecyl-oxy) methyl-15-crown-5 and thiotic acid) were grafted to gold nanoparticles (See Scheme 30). Without addition of Pb (II) cations, aggregation of nanoparticles was observed, due to interparticle hydrogen bonding. By addition of Pb (II) cations, a significant change in color was observed, indicative of the dispersion of the nanoparticles by breaking interparticle hydrogen bonding. A high selectivity of the nanocomposite for Pb (II) cation was also evidenced.

Sensing of alkali metal ions has attracted considerable interest owing to the significance of these metal ions in biology.

**Scheme 31:**
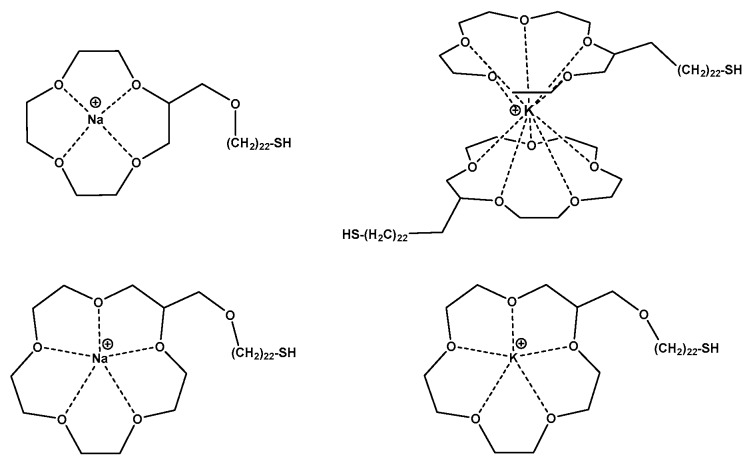
Crown alkali ether used to functionalize Au° nanoparticles.

Among recent approaches to the design and the fabrication of alkali sensors, gold nanoparticles-based sensors have focused much attention as a promising operating principle. By addition of alkali cations, one simple way of signaling recognition events is to generate a modification in the fluorescence intensity or the color of the solution. Chelation of alkali cations is based in all cases on the use of crown ethers, which are known for their unusual properties of forming stable complexes with alkali cations. Among all crown ethers, 15-crown-5 is known to generate highly stable complexes with Na^+^ cation whereas 18-crown-6 prefers K^+^ as cation (See Scheme 31) [216,217].

The first example of colorimetric sensing is based on 15-crown-5-functionalized gold nanoparticles, where the linkage between the crown ether and the particles is insured by a dodecyl chain [218]. Stable particles were thus obtained in water due to steric repulsions between particles. Stable colloids were also obtained upon addition of Na^+^, whereas addition of K^+^ resulted in the aggregation of the particles with a noticeable color change. The stabilization of K^+^ cation being ensured by two crown ethers, the chelation of K^+^ cations by crown ethers involved into two different complexations was observed, resulting in their aggregations. Another group of research took advantage of aggregation by the addition of cations to prepare assembled nanoparticle films [219]. A modification of that first system published by Li was brought by the introduction of a second functionality on the nanocomposite [220]. A cooperative effect was observed when thiotic acid was grafted on the nanoparticles, whereas replacement of thiotic acid by thioctic amine altered the properties. Bifunctionalized nanoparticles were prepared according a two-step procedure consisting first of a ligand exchange of citrate ligand by thioctic acid, followed by a second partial exchange of thiotic ligands by thiolated crown ether [221,222]. Influence of the spacer on the kinetic of complexation was also studied, and a similar study was realized with 12-crown-4 and Na^+^ as a cation [220].

### 7.8. Functionalisation by Coordination Complexes of F-Block Elements

**Scheme 32:**
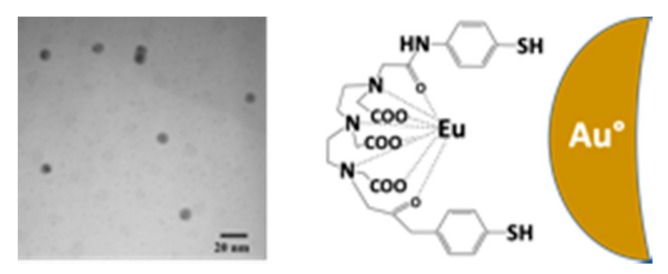
TEM Pictures of Eu(III) complexes grafted onto Au-NPs as described in ref. [204]. Reproduced from [225], RSC 2006.

To date, few examples of lanthanide complexes on gold nanoparticles are reported in the literature [223,224]. Several complexes were covalently grafted on gold nanoparticles due to their luminescent properties. In most of the cases, the goal was to prepare ion sensors. As a first example—and to ensure a strong binding of the complex to the surface of the nanoparticle—a diethylene triamine pentaacetic acid (H_5_DTPA) substituted with two thiophenol groups was used [225]. A water-soluble Eu (III) nanocomposite and luminescent nanobeads were thus obtained (See Scheme 32) [225].

**Scheme 33:**
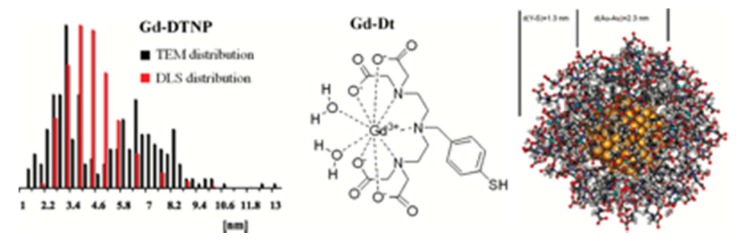
Gd^3+^ complexes used to coat Au-NPs for MRI applications as described in [230]. Reproduced with permission from [230], ACS, 2006.

Another europium complex grafted via a long alkyl chain to gold nanoparticles was used as an efficient sensor for phosphate-anion sensing in aqueous solution [226]. In this case, detection of phosphate anions was done in three steps, the first one consisting in the grafting of a none-luminescent Eu (III) complex. Then, addition of a β-diketone gave rise to a highly luminescent complex, by exchange of two water molecules, with one molecule of β-diketone on the complex. The last step consisted of the addition of a phosphate anion that switched off the luminescence. Eu (III) and Tb (III) complexes were also used as sensors for metal cations [227]. These nanocomposites—based on bipyridine capped nanoparticles—proved to be highly phosphorescent. Especially for Eu (III)-based nanocomposites, addition of earth metal ions and transition metal ions resulted in the decrease of luminescence, due to an isomorphous substitution of Eu (III) ions by these cations. Gd (III) complexes on particles were used in vivo as contrast agents, combining both X-ray-computed tomography and Magnetic Resonance Imaging (MRI) (See Scheme 33) [228,229]. These particles, suitable for dual imaging, exhibited a strong contrast enhancement in MRI stems. Other and recent works described Gd^3+^ complexes for IRM applications [230,231,232].

## 8. Conclusions

To conclude, the diversity of Au°-based nanomaterials comprising inorganic entities as functionalizing agent was clearly evidenced. This unique combination between Au-NPs and these inorganic entities opened the way for Au nanoparticles for practical applications ranging from catalysis, opto-electronic, or biomedical applications. In these different examples, the stability of the resulting nanocomposites is a major issue for further applications and this point is still an active research field. Depending on the applications, the toxicity of the functionalizing agent must also be considered; this point has only been scarcely examined in these different works. Considering that the stability and the toxicity of the nanocomposites will govern their future applications, this point should be carefully examined in the coming years.
